# Pre-notification of arriving trauma patient at trauma centre: A retrospective analysis of the information in 700 consecutive cases

**DOI:** 10.1186/1757-7241-16-15

**Published:** 2008-11-19

**Authors:** Lauri E Handolin, Juhapetteri Jääskeläinen

**Affiliations:** 1 Töölö Hospital, Department of Orthopaedics and Traumatology, Helsinki University Hospital, Topeliuksenkatu 5, FIN-00260 Helsinki, Finland

## Abstract

**Background:**

Pre-notification of an arriving trauma patient, given by transporting emergency medical unit, is needed in terms of facilitating the admitting emergency department to get ready for the patient before the patient actually arrives. In the present study we retrospectively analyzed the pre-hospital information provided by 700 consecutive pre-notification mobile phone calls in terms to asses the response of trauma team activation regard to pre-notified information such as vital signs and level of consciousness, mechanism of injury (MOI), and estimated elapsed time (EET) from the time of pre-notification phone call to arrival.

**Results:**

The median EET was 15 minutes (range 0 – 80 min, interquartile range 10 – 20 min). In 11% of the cases EET was 5 minutes or shorter. 17% of the patients were intubated and ventilated on scene at the time pre-notification phone call took place. The most commonly notified pre-hospitally diagnosed injuries were thoracic in 75 cases (11%), followed by unstable long bone (tibia, femur, humerus) fracture in 66 cases (9%), and abdominal injuries in 32 cases (5%). Trauma team was activated for 61% of 700 pre-notified patients. MOI without clinical symptoms was the reason for team activation in 75% of the cases. In 25% of the cases there were pre-hospitally observed clinical injuries or abnormalities in vital parameters.

**Conclusion:**

Pre-notification phone call is of a crucial importance in organizing every day activities at a busy trauma centre, but it should not take place in too much advance. In any case, a pre-notification phone call, even on short notice, gives emergency department personnel some time to prepare for the incoming patient.

## Background

Effective regionalized trauma care requires establishment of triage criteria that identify the patients who will benefit from the services and resources available at trauma centre. Mortality is associated with undertriage (that is, not to transport all patients to trauma centre who would benefit from it) [1]. On the other hand, overtriage (that is, the transport of patients with minimal injuries to trauma centre), while less threatening from a medical standpoint, may generate unnecessary utilization of trauma centre resources. The American College of Surgeons' Committee on Trauma (ACS-COT) has suggested that a 30 to 50% rate of overtriage may be necessary to maintain an acceptable undertriage rate [2]. In an optimal scenario, patients receive treatment at the appropriate institution, resources are allocated appropriately, and the clinical outcome is optimized [3].

Ambulance-hospital pre-notification of impending arrival of trauma patient to the emergency department (ED) is of crucial importance. Pre-notification gives the ED few minutes to judge the level of needed preparation maneuvers, including the decision whether to activate the trauma team or not. It also facilitates the ED to prepare the practical issues and logistics for arriving trauma patient. The benefit of appropriate pre-notification is documented also in care of stroke patients and acute myocardial infarction patients by shortening the door to medical review and the door to needle time, respectively [4,5].

Helsinki University hospital provides acute trauma care for Helsinki and it's surroundings, resulting in a catchments area of about 1.5 million people (25% of the Finnish population). Töölö hospital, Helsinki University Hospital's trauma centre, provides the acute care for vast majority of major blunt traumas excluding patients younger than 16 years not having a suspected brain injury and patients with a major penetrating torso trauma. Töölö hospital is the largest trauma centre in Finland, and one of the largest in Scandinavia, with annual number of patients having ISS>15 and > 22 being 550 and 350, respectively [6].

The emergency medical system (EMS) provides ambulance-hospital pre-notification for Töölö hospital practically on every arriving trauma patient. The aim of the present study was to analyze the information provided by pre-notifications of arriving trauma patients, and to analyze the response on the trauma team activation (TTA) in regard to varying kind of pre-notified information, such as vital signs (VS) and mechanism of injury (MOI).

## Methods

Regarding the routine Töölö hospital trauma protocol, the information of every pre-notification phone call ED receives, is written down and archived. Pre-notification information is routinely collected on special form developed for the purpose, focusing on the issues related to the MOI, VS and LOC, anatomic injury (AI), and the EET (median, range, interquartile range (IQR)).

During the 12 month period, from September 1^st ^2005 to August 31^st ^2006, all consecutive pre-notification forms of the arriving trauma patients were retrospectively reviewed. The pre-notified and recorded data on MOI, VS, LOC, AI, TTA and EET was analyzed. In terms of EET, the arriving patients were divided into three categories; the ones arriving from inside the city of Helsinki, the ones arriving from two surrounding major cities (Espoo and Vantaa), and the ones arriving from outside of these three cities.

If GCS (Glasgow coma scale) is not assessed on scene, EMS personnel are asked to describe the level of consciousness by "normal", "decreased", or "unconscious". In addition to a clinical description of level of consciousness, the contacting EMS personnel are also asked to asses the hemodynamics as "stable" or "unstable". Both Revised Trauma Score (RTS) [7] and coded RTS of arriving patients were assessed in the present study if all the needed parameters (respiratory rate, systolic blood pressure, and GCS) were available.

The present study focuses only on the crucial information provided by EMS before the arrival of trauma patient. No comparisons to the clinical findings or outcome in hospital were made. Due to the nature of the present study, further statistical analyses were not conducted nor there was a need for institutional board approval.

## Results

During the 12 month study period, the ED at Töölö hospital received 700 pre-notification phone calls on arriving trauma patients (on average 58 calls per month). The high incidence months were July and August, February and March being the "silent" ones. The hourly distribution of pre-notification phone calls was observed to be relative even during the 24 hour period: 31% took place between 07 – 15 hours, 39% between 15 – 22 hours, and 30% between 22 – 07 hours, respectively.

**Figure 1:**
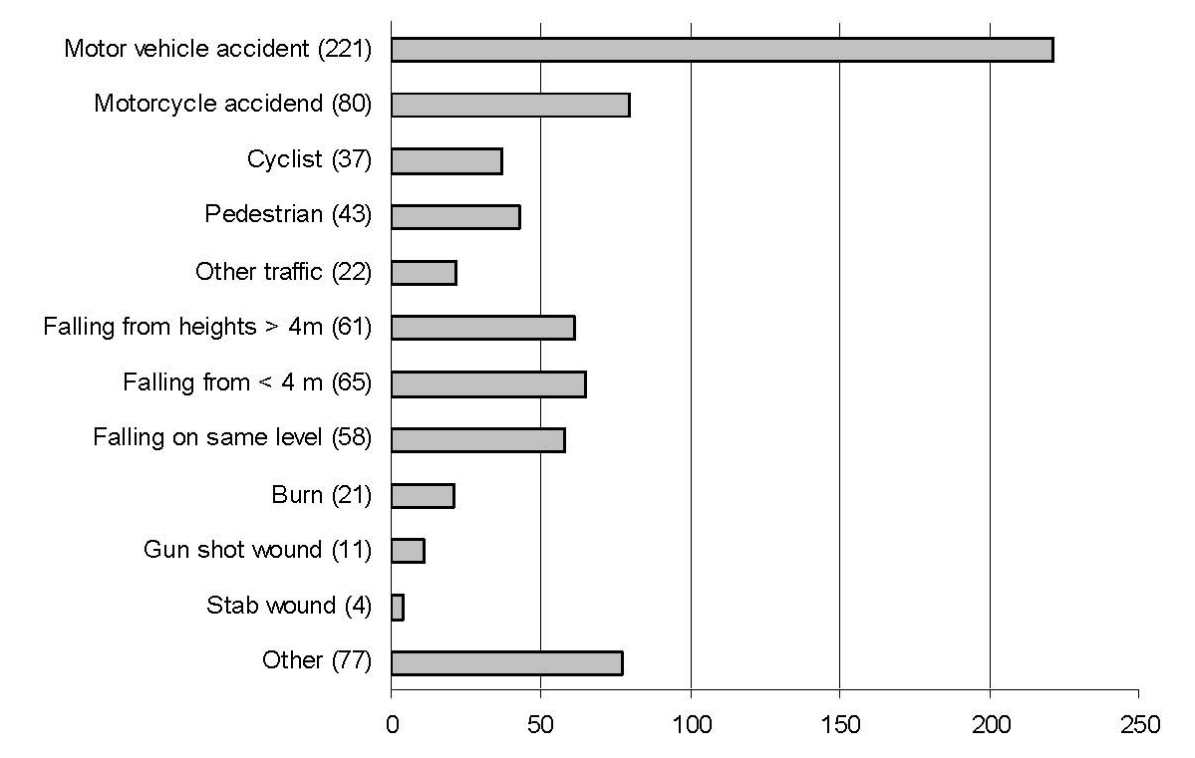
The mechanisms of injuries of 700 pre-notified arriving trauma patients during 12 month study period at Töölö hospital emergency department (number of pre-notifications in brackets).

The median EET from the time of pre-notification phone call to arrival was 15 minutes (range 0 – 80 min, IQR 10 – 20 min) in all patients, but tended to be a little longer (15 min, range 0 – 80 min, IQR 10 – 25 min) in cases the trauma team was judged to be activated. In 11% of the cases EET was 5 minutes or shorter. The median EET was 10 minutes (range 0 – 35 min, IQR 5 – 15 min) in patients coming inside the city of Helsinki (229 patients), and 15 minutes (range 1 – 35 min, IQR 10 – 16 min) in patients coming from surrounding cities of Espoo and Vantaa (144 patients). The longest median EET, 20 minutes (range 0 – 80 min, IQR 15 – 30 min), was observed in patients coming outside of the surrounding cities (327 patients). The mechanisms of injuries are presented in Figure 1. 97% of the pre-notified patients had sustained blunt injury (3% sustained penetrating injury). In 42 cases (6%) EMS pre-notified of two or more (two to five) simultaneously arriving trauma patients from the same injury site.

17% of the patients were intubated on scene at the time pre-notification phone call took place. Also, all the needed information for assessing RTS was present only in 6% of the non-intubated cases. The most common missing RTS parameter was respiratory rate, missing in 94% of the non-intubated cases. In patients with all the needed data, the median RTS was 7.841 (range 5.030 – 7.841, IQR 6.904 – 7.841) and the median coded RTS 12 (range 8 – 12, IQR 11 – 12). Vital signs and level of consciousness at the time of pre-notification phone call in all patients, patients intubated on scene, and patients with TTA is presented in Table 1.

**Table 1:** Median values of vital parameters at the time of pre-notification phone call in all patients, intubated patients, and patients with trauma team activation (range, interquartile range).

	Heart rate	Respiratory rate	Systolic blood pressure	GCS
All patients	90 (43 – 180, 76 – 100)	18 (10 – 50, 15 – 24)	124 (50 – 245, 110 – 140)	15 (3 – 15, 13 – 15)
Intubated on scene	85 (48 – 140, 70 – 100)	-	115 (50 – 210, 100 – 130)	-
Trauma team activated	90 (48 – 180, 78 – 100)	20 (10 – 50, 16 – 24)	120 (56 – 220, 110 – 140)	14 (3 – 15, 6 – 15)

The percentage of pre-notified patients sustaining observed anatomic injuries (AI) was noted to be low. The most commonly notified pre-hospitally diagnosed (or strongly suspected) injuries were thoracic in 75 cases (11%), followed by unstable long bone (tibia, femur, humerus) fracture in 66 cases (9%), and abdominal injuries in 32 cases (5%). Unstable pelvic ring fracture was observed in 10 and amputated extremity in three cases (1% and 0.4%, respectively).

In nine cases (1%) contacting EMS pre-notified of the need for a prompt emergency procedure to be carried out immediately upon arrival. In four cases (0.6%) EMS was not able to establish patent airway, in another four cases (0.6%) there was a need for proper decompressive thoracotomy, and in one case (0.1%) EMS was not able to establish any proper intravenous lines.

There were no remarks on possible TTA in 63 (9%) studied pre-notification forms. In the rest of 637 cases, trauma team was activated for 389 pre-notified patients (61%). MOI was the only reason for TTA in 75% of the cases. The reason to TTA and the person judging the TTA is presented in Table 2.

**Table 2:** Characteristics related to trauma team activation on the basis of information obtained from the 700 pre-notification phone calls.

	no. of cases/all cases	percentage of cases
Trauma team activated	389/637	61%
Reason for trauma team activation recorded	255/389	66%
Reason – abnormal vital signs or level of consciousness	29/255	11%
Reason – mechanism of injury	191/255	75%
Reason – EMS doctor escorting the patient (no abnormal vital signs or level of consciousness)	35/255	14%
Personnel judging trauma team activation recorded	469/700	67%
Judgment of activation – trauma nurse	288/469	61%
Judgment of activation – trauma team leader	146/469	31%
Judgment of activation – trauma nurse and team leader in consensus	35/469	7%

637 (91%) studied pre-notification forms contained sufficient information for the analysis.

## Discussion

A recent study indicates a lower risk of death when care of traumatized patient is provided in a trauma centre compared to non-trauma centre [1]. Different standardized protocols and procedures on trauma care are characteristic routines in a dedicated trauma centre. Pre-notification of an arriving trauma patient is needed for giving an ED some minutes to get ready for the patient before the patient actually arrives.

In the preparing process for an arriving trauma patient at ED, two levels can be identified; basic and special level. In basic level, all the basic preparing procedures, such as trauma team activation, are carried out. This is normally enough for vast majority of arriving trauma patients. However, in some cases EMS meets physiological or anatomical conditions, such as lack of patent airway, which has to be taken care of immediately upon arrival. These pre-notified conditions launch special level of preparations, and are of crucial importance in executing emergency operations promptly after an arrival. However, the pre-notified information should be kept simple and focused only in relevant issues, since only parts of verbal information can be recalled when taking care of arriving trauma patients [8].

The median EET from the time of pre-notification phone call was observed to be 15 minutes in the present study. Our experience in Töölö hospital ED is that 15 minutes is an optimal period of time, since it allows individual trauma team members to work in different parts of hospital still being able to reach trauma bay well before the arriving patient. On the other hand, if pre-notification takes place too much in advance, there is always a risk that individual team members may end up doing something else before entering trauma bay, and thus meeting a risk of being late. It might even be favorable to ask peripherial EMS, the ones bringing patients from outside of the downtown, to give their pre-notifications little later en route in terms of decreasing inappropriate long EETs. On the other hand, future technology, such as global positioning based real-time tracking systems and digital data transmission between EMS and hospitals, could provide us with more accurate and precise pre-notifications in the future.

There were two or more simultaneous patients arriving from the same injury site almost once a week. In addition to that, there might be simultaneous trauma patients arriving from different injury sites resulting in multiple patient scenarios. In such cases, it is obligatory for ED to get pre-notification in terms of recruiting enough personnel to accommodate the needed number of trauma teams. That becomes of crucial importance in scenarios when the number of ED personnel is not enough, and more personnel has to be recruited from the other parts of hospital.

It has been stated that unnecessary trauma team activations should be balanced in terms of gaining optimal initial trauma care to all severely injured patients [9]. That is, trauma teams involving several specialties and personnel are considered expensive and limited resource, which should be utilized in reasonable manner. Also, the efficient use of hospital resources utilized in TTAs, should be addressed in economical points of view. In addition to the disturbance for normal hospital work the team activation results, through its personnel leaving the routine daily tasks and gathering to the trauma bay, the unnecessary utilization of teams may result in decrease of the team morale.

Normally trauma admitting hospitals, including our, base their trauma team activation criteria on three categories including observed physiological signs, anatomical symptoms, and mechanism of injury. In recent rapport from Denmark, a level 1 trauma centre using ACS-COT criteria, the sensitivity (zero undertriage) of that triage protocol was 92%, the specificity (zero overtriage) being 76% [9]. There are studies showing that MOI criteria alone are inadequate to identify those in need of trauma team activation [10,11]. In recent paper from level 1 hospital in Norway, the MOI as a trauma team activation criterion had a sensitivity of 14% and positive predictive value (the probability of serious injury conditional on team activation) of 7% resulting in a 93% overtriage [11].

Coded RTS-methodology is not routinely used by the Finnish prehospital personnel. Thus, it was not a surprise that all the needed parameters for RTS-scoring were present only in 6% of the studied pre-notifications. Our experience is that numerical coded RTS values are not necessarily needed in every day practice but clinical categories, such as "normal or decreased", may serve as appropriate substitutes.

## Conclusion

Pre-notification phone call indicating estimated elapsed time to arrival, physiological condition, and number of arriving trauma patients are of crucial importance in every day activities of a busy trauma centre. In any case, a pre-notification phone call, even on short notice, gives emergency department personnel some time to prepare for the incoming patient.

## Competing interests

The authors declare that they have no competing interests.

## Authors' contributions

JJ gathered the data, participated in analyzing and interpretation the data, and participated in drafting and finalizing the manuscript. LH conceived and designed the study, participated in analyzing and interpretation the data, and drafted and finalized the manuscript. Both authors read and approved the final manuscript.
